# An Optimized Protocol for the Isolation and Functional Analysis of Human Lung Mast Cells

**DOI:** 10.3389/fimmu.2018.02193

**Published:** 2018-10-05

**Authors:** Avinash Ravindran, Elin Rönnberg, Joakim S. Dahlin, Luca Mazzurana, Jesper Säfholm, Ann-Charlotte Orre, Mamdoh Al-Ameri, Peter Peachell, Mikael Adner, Sven-Erik Dahlén, Jenny Mjösberg, Gunnar Nilsson

**Affiliations:** ^1^Immunology and Allergy Unit, Department of Medicine Solna, Karolinska Institutet, Karolinska University Hospital, Stockholm, Sweden; ^2^Department of Medicine Huddinge, Center for Infectious Medicine, Karolinska Institutet, Karolinska University Hospital, Stockholm, Sweden; ^3^Unit for Experimental Asthma and Allergy Research, Centre for Allergy Research, The Institute of Environmental Medicine, Karolinska Institutet, Stockholm, Sweden; ^4^Thoracic Surgery, Department of Molecular Medicine and Surgery, Karolinska Institutet, Karolinska University Hospital, Stockholm, Sweden; ^5^Academic Unit of Respiratory Medicine, University of Sheffield, The Royal Hallamshire Hospital, Sheffield, United Kingdom; ^6^Department of Clinical and Experimental Medicine, Linköping University, Linköping, Sweden; ^7^Department of Medical Sciences, Uppsala University, Uppsala, Sweden

**Keywords:** mast cells, lung, enzymatic digestion protocols, human mast cells, mast cell isolation

## Abstract

**Background:** Mast cells are tissue-resident inflammatory cells defined by their high granularity and surface expression of the high-affinity IgE receptor, FcεRI, and CD117/KIT, the receptor for stem cell factor (SCF). There is a considerable heterogeneity among mast cells, both phenotypically and functionally. Human mast cells are generally divided into two main subtypes based on their protease content; the mucosa-associated MC_T_ (tryptase positive and chymase negative mast cell) and the connective tissue associated-residing MC_TC_ (tryptase and chymase positive mast cell). Human lung mast cells exhibit heterogeneity in terms of cellular size, expression of cell surface receptors, and secreted mediators. However, knowledge about human lung mast cell heterogeneity is restricted to studies using immunohistochemistry or purified mast cells. Whereas the former is limited by the number of cellular markers that can be analyzed simultaneously, the latter suffers from issues related to cell yield.

**Aim:** To develop a protocol that enables isolation of human lung mast cells at high yields for analysis of functional properties and detailed analysis using single-cell based analyses of protein (flow cytometry) or RNA (RNA-sequencing) expression.

**Methods:** Mast cells were isolated from human lung tissue by a sequential combination of **w**ashing, **e**nzymatic digestion, **m**echanical disruption, and density centrifugation using **P**ercoll (WEMP). As a comparison, we also isolated mast cells using a conventional enzyme-based protocol. The isolated cells were analyzed by flow cytometry.

**Results:** We observed a significant increase in the yield of total human lung CD45^+^ immune cells and an even more pronounced increase in the yield of CD117^+^ mast cells with the WEMP protocol in comparison to the conventional protocols. In contrast, the frequency of the rare lymphocyte subset innate lymphoid cells group 2 (ILC2) did not differ between the two methods.

**Conclusion:** The described WEMP protocol results in a significant increase in the yield of human lung mast cells compared to a conventional protocol. Additionally, the WEMP protocol enables simultaneous isolation of different immune cell populations such as lymphocytes, monocytes, and granulocytes while retaining their surface marker expression that can be used for advanced single-cell analyses including multi-color flow cytometry and RNA-sequencing.

## Introduction

Mast cells are inflammatory cells that hold several important roles in health as sentinel cells with multifunctional properties (1). They are one of the main actors in the immune response against pathogens, but also in the recognition of “danger,” i.e., cell injury or cell stress (2, 3). When the cells recognize a pathogen or a danger signal they release mediators that have vascular effects and/or recruits and activates other cells of the immune system (4). The other side of this potent effector cell is its detrimental effects in the pathophysiology of several disorders, including airway diseases (5, 6).

Mast cell progenitors arise from the pluripotent hematopoietic stem cells of the bone marrow and circulate in blood where they can be found in low frequency (7–10). These progenitor cells subsequently migrate into tissues where they mature under the influence of growth factors like stem cell factor (SCF) (11). The tissue-resident mast cells are characterized by their expression of the high-affinity IgE receptor, FcεRI, the SCF receptor KIT/CD117, and their high cellular complexity with granules containing, e.g., histamine, proteoglycans, and proteases. These mature mast cell populations are found in all tissues, but predominantly in the skin and at mucosal surfaces in the respiratory, gastrointestinal, and urogenital tracts (11, 12). Within the tissue they are highly integrated with the extracellular matrix and other tissue resident cells. The fact that mast cells are exclusively tissue resident cells, tightly bound to the tissue, makes their isolation process challenging.

Mast cells display great phenotypic and functional heterogeneity in terms of expression of surface markers, granular content, responsiveness, and activation via different pathways (13). Human mast cell phenotypes are classically divided into two main types based on their protease content (14). Tryptase-positive mast cells, designated as MC_T_, are predominantly found in mucosal surfaces such as within the bronchial airway epithelium. MC_TC_ cells contain, in addition to tryptase, also chymase, carboxypeptidase A, and cathepsin G like protease and is the predominant type in connective tissues such as the skin (15). Mast cell phenotypes not only vary greatly between different organs but also between different sub-compartments of an organ. Detailed immunohistochemical characterization of human lung mast cells demonstrated their heterogeneity within the subcompartments of human lung (16). However, a detailed characterization of the intra-cellular variation among human lung mast cells remains to be deciphered.

Mast cells are implicated in the pathogenesis of several airway diseases such as asthma, chronic obstructive pulmonary disease, interstitial pulmonary fibrosis, and pulmonary hypertension (5, 17–19). The release of granular mediators, lipid mediators and cytokines, and their close proximity to nerve endings, airway smooth muscle cells, epithelial cells, endothelial cells, and immune cells explains the profound effects that mast cells can have on airway pathophysiology (6). Characterization of lung mast cell heterogeneity and the regulation of their function under homeostatic and disease conditions is crucial for the understanding of their contribution to the pathology in these diseases. Several studies have been published where human lung mast cells have been purified using different types of approaches, e.g., enzymatic dispersion, elutriation, percoll gradient centrifugation and magnetic cell separation, and the function of the cells have been analyzed (20–24). Nevertheless, there is a need for an efficient method to isolate mast cells at high yield from the lung tissue to be able to perform in depth characterization of human lung mast cells at the single cell level. Methods like multidimensional cytometry (flow cytometry or mass cytometry/CyTOF) and single-cell RNA-sequencing on isolated human lung mast cells will be key in revealing the role for mast cells in airway diseases. This paper describes a protocol that permits isolation of viable mast cell populations with high yield from human lung tissue, which will facilitate further characterization studies.

The protocol is based on four sequential main steps—Wash, Enzymatic digestion, Mechanical disruption, and Percoll-gradient centrifugation (WEMP). The conventional protocols rely mainly on enzymatic digestion, which have lower yield and would not necessarily liberate all the cells from inner compartments of the tissue (25). In this paper, we show that the WEMP protocol efficiently isolates immune cells at higher yield than the conventional protocol, especially of tissue resident mast cells. In addition to increasing the yield of isolated immune cells, the WEMP protocol enables subsequent detailed characterization of cells using flow cytometry and single-cell RNA-sequencing.

## Methods

### Lung tissue

Lung tissue was obtained from tumor patients who underwent surgical lung resection at Karolinska University Hospital, Solna, Sweden. Patients have not received chemotherapy or radiotherapy. All donors participating in the study gave informed written consent, and the study was approved by regional review board in Stockholm.

### Processing of human lung tissue

A small portion of human lung tissue (3–12 g) was cut out from distal region of tumor site. The tissue was immediately transferred into ice-cold Krebs-Henseleit buffer and stored on ice. Tissues were processed using two different protocols.

### Conventional protocol

The human lung tissue was washed with PBS and cut into small pieces and enymatically digested for 45 min in RPMI with DNase I and Collagenase at 37°C with stirring. After enzymatic digestion, cold RPMI with 10% FCS (Fetal calf serum), 4.1 mM L-Glutamine and 1% penicillin/streptomycin (stop media) was added to stop the enymatic process. Cell suspension was passed through a 100 μm filter and washed with stop media and PBS with 2% FCS. Red blood cells were lysed using 5 ml of ACK (ammonium-chloride-potassium) lysing buffer for 5 min on ice and washed with PBS 2% FCS. Cell suspension was resuspended in PBS 2% FCS.

### The washing, enzymatic digestion, mechanical disruption, and percoll—WEMP—protocol

Detailed information about the WEMP protocol follows under stepwise protocol —WEMP-protocol section. In short, human lung tissue was washed with PBS and cut into small pieces that was enzymatically digested for 45 min in RPMI with DNAse 1 and collagenase at 37°C with stirring. Cold stop media was added. Enzymatically digested lung tissue pieces were cut using scissors and mechanically disrupted using a syringe for 10 times and filtered using 100 μm filter and repeated twice. Filtered cell suspension was washed with PBS and resuspended in 30% Percoll (one phase) and centrifuged at 780 g for 12 min. Cell pellet was resuspended in PBS with 2% FCS. Red blood cells were lysed using 5 ml of ACK (ammonium-chloride-potassium) lysing buffer for 5 min on ice and washed with PBS 2% FCS. Cell suspension was resuspended in PBS 2% FCS.

### Flow cytometry analysis and cell sorting

For surface staining of mast cells, the following antibodies were used: APCCy7-CD14 (Biolegend, clone M5E2), PE-FcεRI (Biolegend, clone CRA1), and APC-CD117 (BD Biosciences, clone 104D2), V500-CD45 (BD Biosciences, clone HI30). For surface staining of innate lymphoid cells type 2 (ILC2), the following antibodies were used: FITC- conjugated CD3 (Biolegend, clone SK7), CD19 (BD Biosciences, clone 4G7), CD14 (Dako, clone TUK4), CD1a (Biolegend, clone HI149), CD123 (Biolegend, clone 6H6), BDCA2 (Miltenyi Biotech, clone AC144), TCR α/β (Biolegend, clone IP26), TCR γ/δ (Biolegend, clone B1), CD 94 (Biolegend, clone DX22), PECy7-CD127 (Beckman Coulter clone R34.34), PECy5.5-CD117 (Beckman Coulter, clone 104D2D1), V450-CRTH2 (CD294) (BD Biosciences, clone BM16), PECF594-CRTH2 (BD Biosciences, clone 563501), APC-NKG2A (Beckman Coulter, clone Z199), BV605-CD161 (Biolegend, clone HP-3G10), and V500-CD45 (BD Biosciences, clone HI30). For single cell sorting, following antibodies were used along with above mentioned ILC2 antibodies: BV711-CD56 (Biolegend, clone HCD56), PECy5-Nkp44 (Beckman Coulter, clone Z231), AF700-CD16 (Biolegend, clone 3G8), Invitrogen Live/Dead™ Fixable Green Dead Cell Stain kit. FlowJo software was used for flow cytometry data analysis.

### Intracellular staining of tryptase

Human lung cells (prepared using the WEMP-protocol) where stained using V500-CD45 (clone HI30), APC-Cy7-CD14 (clone M5E2), PE-CD117 (clone YB5.B8), BD Biosciences, thereafter fixed with 4% PFA and permeabilized using PBS-S buffer (0.1% saponin in PBS with 0.01 M HEPES). Unspecific binding was blocked using blocking buffer (PBS-S with 5% dry milk and 2% FCS). Cells were thereafter stained with Tryptase antibody (Millipore, clone G3) conjugated in house with Alexa Flour 647 (Alexa Flour 647 Monoclonal antibody labeling kit, Invitrogen).

### Activation of human lung mast cells by FcεRI crosslinking

Single cell suspensions derived from human lung tissue obtained by the WEMP-protocol were cultured at a density of 2 million cells/ml in complete RPMI-1640 medium (Sigma-Aldrich, St. Louis, MO, USA) supplemented with 10% FCS (Life Technologies, Paisley, UK), 100 ng/ml SCF (Sobi, Stockholm, Sweden) and cultured for 4 days. For activation, cells were incubated with 1 μg/ml IgE (Calbiochem, Merck Millipore, Darmstadt, Germany) overnight and activated using 2 μg/ml α-IgE (Sigma, St Louis, MO) for 30 min. Cells were then stained using BD Horizon™ Fixable Viability Stain 450 and antibodies CD45 (BD Biosciences, clone HI30), CD14 (Biolegend, clone M5E2), CD117 (BD Biosciences, clone 104D2), CD63 (BD Biosciences, clone H5C6) and mast cells were gated as live, CD45+, CD14–, CD117 high and activation was determined by CD63 translocation to the membrane.

### Materials required

Stop media (cold): 10% FCS (Gibco), 4.1 mM L-Glutamine (Sigma) and 1% penicillin/streptomycin (Sigma), in 500 ml of sterile RPMI media (GIBCO).

Enzyme buffer: 3 ml of 1 M HEPES (Sigma) in 200 ml of sterile RPMI media (GIBCO) (pH 7.4). Wash buffer: PBS. FACS buffer: PBS with 2% FCS. Digestion enzymes with final concentrations of 0.125 mg/ml (0.5–5 units/mg) collagenase Type II (Sigma) and 0.2 mg/ml (2,993 units/mg) DNase I (Roche) in enzyme buffer. Percoll (GE healthcare, Uppsala, Sweden).

PBS 10x, 1x. RPMI and PBS are kept in sterile condition throughout the experiment.

Plastics: 100 × 15 mm petri dishes (Falcon), 50 ml polypropylene tubes (Falcon), 50 ml polypropylene tubes (Falcon), scissors, forceps, scalpel, 100-μm cell strainers, 50 and 100 ml beakers, pipettes, pipette tips, magnetic stirrer, ice box, and ice. Tabletop centrifuge, laminar airflow chamber, cells counter, weight scale, and a beaker for water bath.

### Statistical analysis

Statistics were calculated using Graphpad Prism. Unpaired 2-tailed *t*-tests were used when comparing 2 groups and one-way analysis of variance (ANOVA) with Bonferroni's multiple comparison test was used when 3 or more groups were compared. *P* < 0.05 is considered significant.

### Stepwise procedure

#### WEMP-protocol

**Washing and processing tissue**

**Figure 1:**
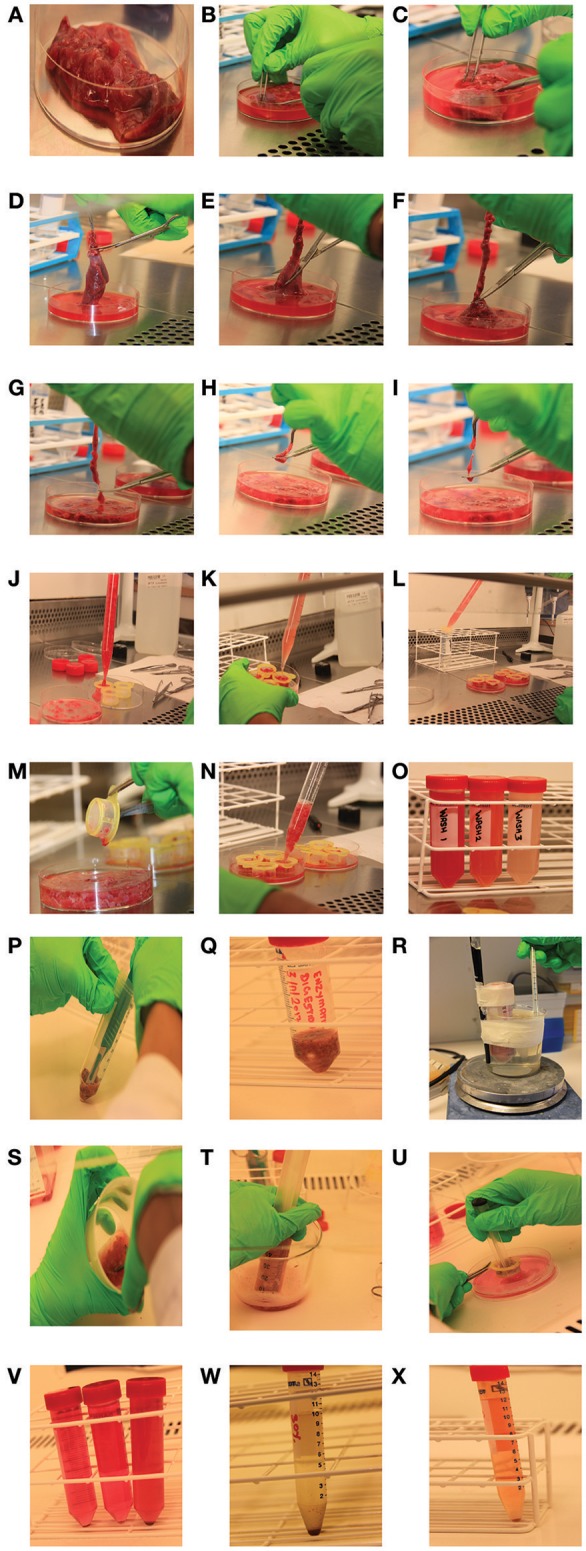
Human lung tissue processing: pictures taken during different steps of WEMP protocol. **(A–C)** Washing tissue, removing blood pockets. **(D–I)** Cutting tissue into thin strips and then into small pieces. **(J–O)** Washing and filtering uniformly cut pieces with PBS. **(P)** Processing tissue pieces with scalpel. **(Q,R)** Enzymatic digestion of tissue pieces at 37°C with magnetic stirrer. **(S–V)** Mechanical disruption of digested tissue using syringe. **(W,X)** Percoll gradient centrifugation and RBC lysis.

1. Tissue piece is transported from the surgery room in Kreb's buffer on ice. Transfer the piece to a 100 × 15 mm sterile petri dish (Figure 1A).
2. Weigh the tissue.
3. Add 50 ml PBS to the petri dish containing the tissue piece. Gently press tissue with forceps and remove red blood cells and larger blood pockets (Figures 1B,C).
4. Cut the tissue into thin uniform strips (as long as possible) (Figures 1D–F) and then each strip into small pieces (0.5 cm) (Figures 1G–I).
5. Wash tissue through a 100 μm cell strainers in a petridish to remove red blood cells (Figures 1J–L). Discard wash (or keep it to analyze loosely bound cells as shown in Figure 1O).
6. Put the filtered tissue pieces back in a petridish, add 50 ml of PBS to filtered tissue pieces in a petri dish (Figures 1M,N). Control that the pieces are uniformly cut, cut any bigger pieces (Figures 1J–L).
7. Repeat step 5–6 twice more.
8. Collect filtered tissue pieces and weigh the tissue again.

**Enzymatic digestion**

9) Place the tissue in a 50 ml tube and cut it finely using scalpel (Figure 1P).
10) Add 1 ml of pre warmed enzyme buffer per gram of tissue (add a minimum 5 ml of enzyme buffer for tissues weighing below 5 g).
11) Add collagenase (0.125 mg/ml of enzyme buffer) and DNase I (0.2 mg/ml of enzyme buffer) (Figure 1Q).
12) Transfer the tube to a pre-warmed water bath at 37°C and stir the content using a magnetic stirrer for 45 min (Figure 1R) (NOTE: After digestion, the tissue solution should appear murky. If the tissue is very fibrotic all the small pieces stick together after this step).
13) Remove the tube from the water bath and add 25 ml of cold stop media (RPMI + 10% FCS + 4.1 mM L-glut + 1% pen/strep) to stop the digestion.

**Mechanical disruption**

14) Collect cell suspension with digested tissue pieces in a small plastic container (~500 ml). Cut the digested tissue finely using 3 scissors (hold 3 scissors in same hand and simultaneluosly use it for cuttting tissues) to disrupt all small tissue pieces that have stuck together (Figure 1S).
15) Plunge the tissue ~10 times with a 50 ml syringe (Figure 1T) & (Supplementary Video 1) [NOTE: If tissue is bigger than 10 grams, divide it into two separate containers for mechanical disruption step. Tips if the process is too tough are (1) do not press the syringe against the bottom of the beaker (2) cut the pieces smaller (3) add more fluid (4) use a smaller (20 ml) syringe and take it in smaller steps].
16) Filter the cell suspension (100 μm) using 3–4 filters in a petridish and collect filtered media in a 50 ml falcon tube.
17) Put the filtered tissue pieces back in the plastic container, add 40–60 ml of stop media and repeat step 14–17 twice more (Figures 1S,T).
18) Squeeze the tissue in a 100 μm filter with flat side of 5 ml syringe (Figure 1U).
19) Spin collected 150 ml cell suspension in 3 falcon tubes (50 ml) at 400 g, 10 min, 4°C (Maximum break) (Figure 1V).
20) Discard supernatants and resuspend cell pellets in stop media. Pool resuspended cell suspension together and make it upto 50ml in stop media and spin at 400 g, 10 min, 4°C (Maximum break).
21) Re-suspend cells in PBS and spin 400 g, 10 min, 4°C (maximum break).

#### Purification of cells

**Percoll gradient centrifugation**

22) Prepare isotonic Percoll (1 ml of 10x PBS + 9 ml of Percoll).
23) Prepare 30% Percoll using 1X PBS (3 ml of Isotonic Percoll + 7 ml of 1x PBS).
24) Re-suspend cells in 10 ml of 30 % Percoll (in 15 ml Falcon tube).
25) Spin 780 g for 12 min with maximum brake at room temperature (Figure 1W).
26) Discard supernatant and wash pellet with 10 ml of PBS 2% FCS. Spin 400 g, 10 min at 4°C with maxium break.
27) Discard supernatant and resuspend cells in 5 ml of 1X ACK lysis buffer to lyse RBC, incubate 5 min on ice.
28) Transfer cells resuspended in ACK lysis buffer to 50 ml falcon tube and add 45 ml of 2% FACS buffer and spin 400 g, 10 min (Figure 1X).
29) Discard supernatant and re-suspend cells in 2% FACS buffer and count the cells.

### Critical parameters and trouble shooting

The efficiency of the whole process primarily depends on the health of the patient (i.e., smoker or non-smoker) and the quality of the speciemen obtained (how fibrotic the tissue is). It is important to pre-warm the enzyme buffer and to set up the water bath in advance, in order to not delay the digestion process. The WEMP protocol takes ~4–5 h (this timing depends on quality and size of tissue).

## Results

### Identification of mast cells in cell suspensions isolated using the WEMP protocol

**Figure 2:**
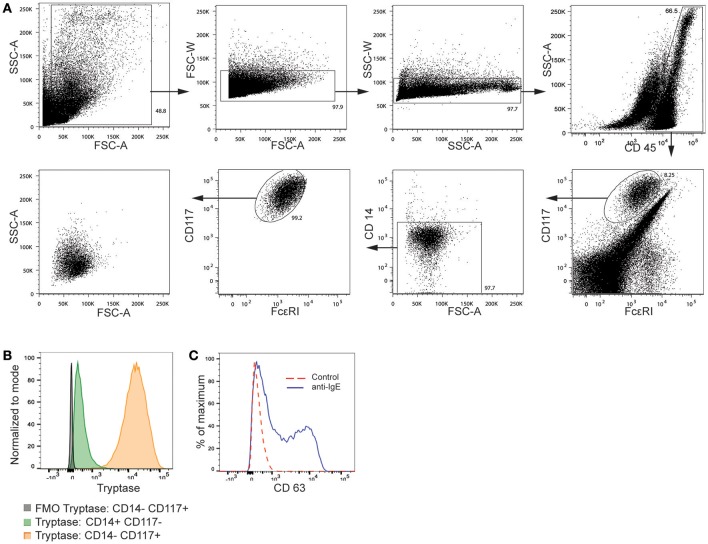
Gating strategy for mast cells from human lung tissue. **(A)** CD117 high mast cells from human lung single cell suspension was identified using flow cytometry. **(B)** CD117 high mast cells and CD14+ cells were analyzed for intracellular Tryptase using flow cytometry. **(C)** Human lung mast cells cultured for 4 days, activated by FcεRI crosslinking and CD63 expression analyzed by flow cytometry. FSC, forward scatter; SSC, side scatter.

The cells isolated according to the WEMP protocol were analyzed by flow cytometry using key surface markers of mast cells. To analyze leucocytes, we first gated on cells expressing CD45. A proportion of the CD45^+^ population were found to express FcεRI and high levels of CD117 and subsequently gated as mast cells. We separately removed cells expressing CD14 (Figure 2A). Confirming their identity as mast cells, the cells expressed intracellular tryptase (Figure 2B). Isolated human lung cells were cultured for 4 days and thereafter activated by anti-IgE for 30 min. The cells were stained and mast cells gated as live, CD45^+^, CD14^−^, CD117^high^. Figure 2C shows surface CD63 of control mast cells (dashed red line) and anti-IgE activated mast cells (dashed blue line). Mast cells from 3 donors were FACS sorted and the RNA sequenced. Transcripts for the mast cell signature genes TPSAB1, HDC, KIT, HPGDS, FcεRIA were all high compared to housekeeping genes (GAPDH). The transcript factor MITF expressed by mast cells was also high compared to GATA3 (not expressed by mast cells), further confirming the identity of the cells as mast cells (Table 1).

**Table 1:** RNA sequencing of sorted human lung mast cells.

**Gene name**	**HLMC #1**	**HLMC #2**	**HLMC #3**
TPSAB1	97,201	40,330	95,685
HDC	63,068	93,662	134,479
KIT	57,019	109,227	106,092
HPGDS	21,767	25,797	27,459
FcεRIA	13,199	13,932	11,755
MITF	3,920	5,228	3,678
GATA3	1	1	3
GAPDH	6,121	5,354	6,089

*Transcript numbers of five mast cell associated genes (TPSAB1, HDC, KIT, HPGDS, and FcεRIA), two transcription factors [MITF (expressed by mast cells) and GATA 3 (not expressed by mast cells)] and one house keeping gene (GAPDH), analyzed in three separate FACS sorted human lung mast cell populations (HLMC) 1–3*.

### Mast cell yield in the conventional and WEMP protocol

**Figure 3:**
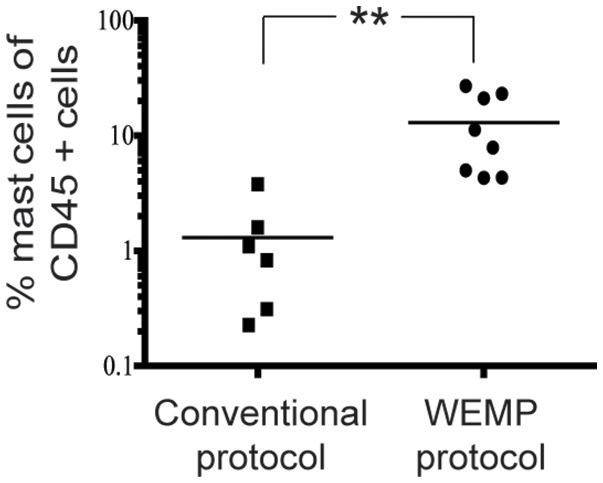
Mast cell yield in Conventional and WEMP protocol. CD45+ CD117 high expressing FcεRI+ mast cell yield compared between conventional and WEMP protocol. **P < 0.01.

We next compared the yield of CD117^high^ FcεRI^+^CD14^−^ mast cells isolated with the WEMP protocol as compared to the conventional protocol. Expressed as a frequency of CD45+ cells, the WEMP protocol was found to give a significantly increased frequency of CD117^high^ FcεRI^+^CD14^−^ mast cells, 10 times higher frequency as compared to the conventional protocol (Figure 3).

### Mast cell yield during the different steps of WEMP protocol

**Figure 4:**
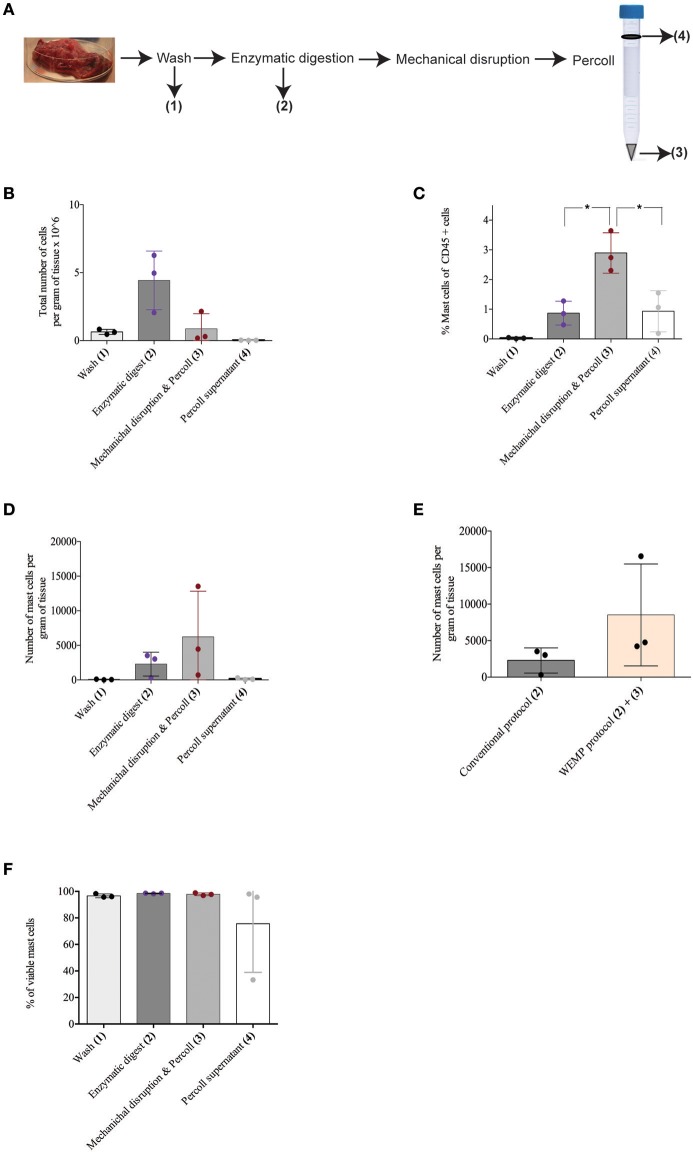
Human lung mast cell yield during different steps of protocol. **(A)** Cells isolated and frequency, number of mast cell analyzed from different steps of the protocol—WEMP. **(B)** Total number of cells from different steps of the protocol counted using microscope. Cells were collected from different steps of the protocol—wash, enzymatic digest, cell pellet and supernatant after mechanichal disruption followed by gradient centrifugation (Percoll purification). **(C)** Mast cells of CD45+ population from different steps of the protocol analyzed by flowcytomtery. **(D)** Number of mast cells per gram of tissue from different steps of protocol counted using microscope. **(E)** Number of mast cells per gram of tissue compared between conventional and WEMP protocol counted using microscope (by combining different steps of protocol). **(F)** Percentage of viable mast cells during different steps of the protocol analyzed by flowcytometry. *P < 0.05.

The reasons for the improved mast cell yield with the WEMP protocol was further dissected by analyzing the mast cell yields from different steps of the protocol (Figure 4A). Single cell suspensions were collected after four different steps of the protocol (Figure 4A), and red blood cells were lysed using ACK lysing buffer. The percentage mast cells, expressed as percent of CD45^+^ cells, increased following the enzymatic digestion (Figure 4A, step 2, Figure 4C) and even more so after the mechanical disruption followed by Percoll (Figure 4A, step 3; Figure 4C). The frequency and total number of mast cells isolated from the wash and Percoll supernatants was relatively low (Figures 4B,C). However, in contrast to the relative enrichment of mast cells, which was highest following mechanical disruption and Percoll (Figure 4A, step 3, Figure 4C), the total yield of CD45^+^ cells per gram tissue was highest following the enzymatic digestion (Figure 4A, step 2, Figure 4B). A similar trend was observed in percentage mast cells of CD45+ cells and total number of mast cells per gram of tissue for different steps of the WEMP-protocol (Figures 4C,D). Comparing the conventional and WEMP protocol there was a tendency of more mast cells with the WEMP-protocol although it did not reach statistical significance (Figure 4E). The viability of mast cells obtained during different steps was consistently high (Figure 4F). Importantly, almost 100% of the CD45+ cells were viable after isolation as determined flow cytometry (Supplementary Figure 1), indicating that the mechanichal disruption does not affect viability and hence the cells are suitable for functional studies.

### ILC2 isolation using the WEMP protocol

**Figure 5:**
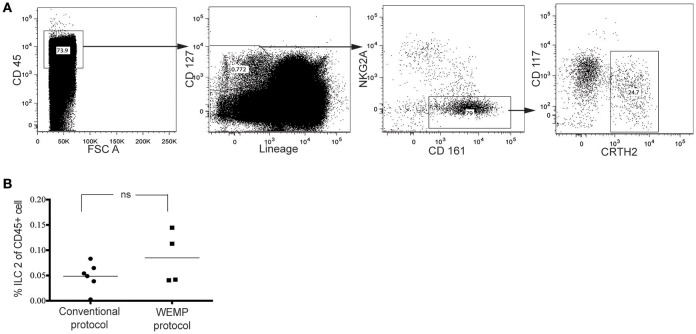
Frequency of ILC2 from human lung tissue. **(A)** Single cell suspension from human lung tissue was stained with markers for lineage, CD45, CD127, CD161, NKG2A, and CRTH2. ILC2 were identified by flow cytometry analysis. **(B)** ILC2 yield was compared between conventional and WEMP protocol.

As the mechanical disruption step potentially improves the isolation of tissue-resident mast cells, we investigated if the WEMP protocol also increased the purification of other tissue immune cells, primarily recruited to the tissue. We were particularly interested in group 2 innate lymphoid cells (ILC2) as this population has been described as tissue-resident in the mouse (26), and also represents a rare lymphocyte subset in the lung. ILC2 was identified as CD45^+^Lin^−^CD127^+^CD161^+^NKG2A^−^CRTH2^+^ cells (Figure 5A) (27).

The frequency of ILC2 among CD45^+^ cells was similar after isolation with the conventional and the WEMP protocol (Figure 5B), indicating that ILC2 are less strongly associated to the tissue as compared to mast cells. Furthermore, these data show that the WEMP protocol is suitable for simultaneous isolation of mast cells and rare, presumably tissue-resident, lymphocyte subsets such as ILC2.

### Analysis of single cell RNA quality

**Figure 6:**
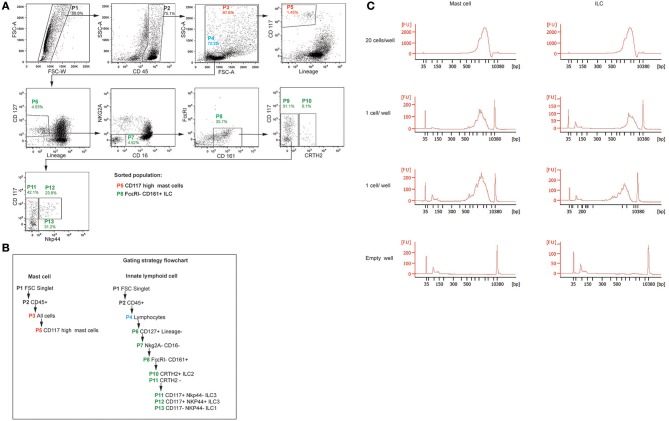
Analysis of single cell RNA quality. **(A)** Gating strategy for sorting mast cells, ILC. **(B)** Gating strategy explained as flowchart. **(C)** RNA quality of single cell sorted mast cells and ILC shown as FU.

Lung tissues were processed using the WEMP protocol, and single cell suspensions were stained for mast cells, ILCs and NK cells, and sorted on the same day as the surgery was performed. Cells were gated for mast cell and ILCs cells as shown in (Figures 6A,B) (28). Single cells were sorted and 20 cells/well was used as a positive control and empty well as negative control. Cells were directly sorted into lysis buffer. The cDNA quality was analyzed by agilent high sensitivity DNA assay as previously described (28) and shown as FU (flourescence units). As shown in Figure 6C the cDNA quality of cells is good using WEMP protocol. Thus, the WEMP protocol is suitable to prepare lung mast cells for single cell RNA sequencing.

## Discussion

In this study we decribe the development of a protocol for the optimization of human lung mast cell isolation. Mast cells are tissue-resident cells tightly bound to the tissue matrix and other tissue-resident cells that explains why a more thorough and robust protocol is needed to obtain a high yield of mast cells residing in different compartments of the lung. The specific protocol that was developed used a combination of enzymatic and mechanical disruption of the tissue, followed by a Percoll density seperation, i.e., the WEMP protocol, generated a high yield of lung mast cells. These cells were functional and the RNA quality was high, thus permitting single cell RNA sequencing.

The WEMP protocol consists of four important steps, wash, enzymatic digestion, mechanical disruption, and Percoll-gradient centrifugation. It is ordered in a way that minimizes cell loss during each step. First step of WEMP protocol—the initial wash, is done gently using forceps to remove blood pockets in tissue and loosely bound red blood cells minimizing number of immune cells lost during wash. In WEMP protocol, reducing the enzymatic digestion step to 45 min ensures that the cell surface markers are not affected for flow cytometry analysis. Mechanical disruption of enzymatically digested lung tissue increases the total number of mast cells isolated, compared to enzymatic digestion alone. Hence, we suggest that WEMP protocol not only increases yield but also helps in the isolation of mast cells from all layers of the human lung tissue, unlike the conventional protocol where mast cells from inner layers of the lung tissue pieces might be missing. The 30% Percoll-gradient centrifugation facilitates the isolation of lymphocyte, monocyte, and granulocyte populations, and importantly removes cell debris.

Our protocol is also efficient for purification of other cell types such as lymphocytes, granulocytes, and dendritic cells using 30% Percoll density centrifugation. However, conventional Ficoll density centrifugation or two phase (30–70%) Percoll density centrifugation can be used for lymphocyte or granulocyte population, respectively, depending on experimental conditions and study of interest. The mechanical disruption and two phase (30–70%) Percoll purification followed by CD117 magnetic sorting will result in mast cell enriched cell suspension (29). However, using a two phase (30–70%) percoll purification of mast cells might lead to the loss of mast cells with particualrly high density/granularity and when investigating the heterogenity of a cell type it is important to not exclude particular fraction of the cells (30).

The percentage of ILC2 of the CD45+ population did not significantly increase using the WEMP protocol compared to the conventional protocol. This is consistent with the fact that mechanical disruption is highly specific for tissue resident cells. However, increase in total number of cells from mechanical disruption will increase isolation of desired cell population. This will be useful in studying rare cell populations such as ILCs.

The localization of different mast cell phenotypes in different compartments of the lung and how this change in airway diseases such as asthma, suggest a possible role of these different mast cell phenotypes in the pathogenesis of asthma. Studies evidently demonstrate the existence of novel human lung mast cell subpopulations with various density, distribution and molecular expression in asthma, IPF, and COPD patients (16, 19, 31). These and other findings support the fact that there is a need for a deeper knowledge for deciphering of mast cell heterogeneity to understand of their roles in the pathology of respiratory diseases. Our developed protocol, WEMP, provides efficient purification of human lung mast cells that can be used for single cell phenotyping, proteomics and RNA sequencing and also functional studies. We believe that this protocol will be very useful for future research in this area.

## Ethics statement

The study was approved by regional review board in Stockholm (dnr. 2010/181-31/2). All subjects gave written informed consent in accordance with the Declaration of Helsinki.

## Author contributions

AR involved in conception and designed of study, performed experiments, analyzed data, and wrote manuscript. GN involved in conception and design of study, wrote manuscript. JM involved in conception and design of study, wrote manuscript. ER, JD, and LM planned and performed experiments. JS, A-CO, and MA-A provided clinical samples. PP, MA, and S-ED revised manuscript. All authors contributed to manuscript writing.

### Conflict of interest statement

The authors declare that the research was conducted in the absence of any commercial or financial relationships that could be construed as a potential conflict of interest. The reviewer NG and handling Editor declared their shared affiliation.

## Supplementary material

Supplementary Figure 1 Cells isolated using WEMP protocol. Percentage of viable cells of CD45+ singlet cells gated.

Supplementary Video 1 Mechanical disruption using 50 ml syringe. Enzymatically digested human lung tissue pieces mechanically disrupted using 50 ml syringe.

## Abbreviations

SCF: Stem cell factor
WEMP: Washing-Enzymatic Digestion-Mechanical Disruption-Percoll purification
ILC2: group 2 innate lymphoid cells
PGD_2_: Prostaglandin D_2_
LT: Leukotrines
MC_T_: Mast cell Tryptase
MC_TC_: Mast cell Tryptase & Chymase
HLMC: Human lung mast cell.
