# Supplementary figures and images for: An Optimized Protocol for the Isolation and Functional Analysis of Human Lung Mast Cells
Source: Front Immunol. 2018 Oct 5;9:2193. doi: 10.3389/fimmu.2018.02193 (PMC6183502; doi:10.3389/fimmu.2018.02193)

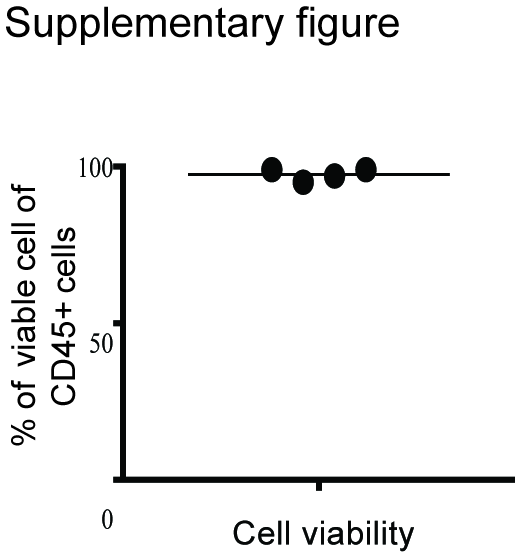

Supplement: Supplementary Figure 1 — Cells isolated using WEMP protocol. Percentage of viable cells of CD45+ singlet cells gated. [file Image_1.TIF]
